# Impaired processing speed in categorical perception: Speech perception of children who stutter

**DOI:** 10.1371/journal.pone.0216124

**Published:** 2019-04-26

**Authors:** Mehdi Bakhtiar, Caicai Zhang, So Sze Ki

**Affiliations:** Department of Chinese and Bilingual Studies, The Hong Kong Polytechnic University, Hong Kong SAR, China; Vita-Salute San Raffaele University, ITALY

## Abstract

There have been controversial debates across multiple disciplines regarding the underlying mechanism of developmental stuttering. Stuttering is often related to issues in the speech production system; however, the presence and extent of a speech perception deficit is less clear. This study aimed to investigate the speech perception of children who stutter (CWS) using the categorical perception paradigm to examine their ability to categorize different acoustic variations of speech sounds into the same or different phonemic categories. In this study, 15 CWS and 16 children who do not stutter (CWNS) completed identification and discrimination tasks involving acoustic variations of Cantonese speech sounds in three stimulus contexts: consonants (voice onset times, VOTs), lexical tones, and vowels. The results showed similar categorical perception performance in boundary position and width in the identification task and similar d' scores in the discrimination task between the CWS and CWNS groups. However, the reaction times (RTs) were slower in the CWS group compared with the CWNS group in both tasks. Moreover, the CWS group had slower RTs in identifying stimuli located across categorical boundaries compared with stimuli located away from categorical boundaries. Overall, the data implied that the phoneme representation evaluated in speech perception might be intact in CWS as revealed by similar patterns in categorical perception as those in CWNS. However, the CWS group had slower processing speeds during categorical perception, which may indicate an insufficiency in accessing the phonemic representations in a timely manner, especially when the acoustic stimuli were ambiguous.

## Introduction

Stuttering is a speech fluency deficit that is mainly manifested by involuntary repetition and prolongation of sounds and syllables, as well as momentary blocks in speech production, which can induce negative impacts on the social, occupational, and academic life of people who stutter (PWS) [1]. Earlier epidemiological studies have reported that 70% of children who stutter (CWS) naturally outgrow their stuttering [2], whereas more recent studies have suggested a higher rate of natural recovery, close to 90%, and the remaining 10% will experience persistent stuttering across their life span [3]. The underlying mechanisms of persistent stuttering have been debated based on many theories, including speech motor control [4] and psycholinguistic theories [5].

Although stuttering is a speech problem mainly manifested in speech production, the presence of an auditory perception deficit and its relevance to speech production is less clear. It has been hypothesized that PWS have a higher reliance on auditory feedback control during speech production due to poorer preparation of feedforward motor commands [4]. Evidence from previous epidemiological studies has shown that the prevalence of stuttering in the hearing-impaired population (0.3–0.4%) is lower than the hearing population (about 1%) [6,7]. Moreover, it has been shown that PWS speak more fluently while speaking under various altered auditory feedback conditions, including masked auditory feedback (i.e., not able to hear one’s own speech), external auditory feedback (e.g., choral speech and speaking in unison with a model speaker) [8,9], delayed auditory feedback [10], and altered auditory feedback (i.e., changing the pitch contour of the auditory feedback) [11,12]. Interestingly, the effect of altered auditory feedback on speech fluency is present even in fast speech production, which suggests that the observed effect is independent of the individual’s speech rate [10].

Empirical studies that have used different paradigms to examine auditory perception in PWS have reported inconclusive results. For instance, some studies that used a nonword repetition task reported poorer performance (i.e., slower reaction times, RTs, and/or higher phoneme errors) in the CWS compared with the CWNS [13–16] and the children who have recovered from stuttering [17], while others reported that the observed differences were not statistically significant [18–20]. Some studies used the phoneme monitoring during an auditory perception task found no significant differences between adults who stutter (AWS) and the matched control group [21]. Along a similar line, some studies that used the phoneme elision task in which the children were required to listen to nonword stimuli and repeat them with a missing sound, found no significant differences in terms of phoneme accuracy and speech initiation time between the CWS and the children who do not stutter (CWNS) [22].

One similarity between the aforementioned studies is that the auditory stimuli were presented within the context of word or nonword stimuli that retained all their contextual cues, which may have activated top-down cognitive processes such as semantic cues and influenced bottom-up auditory perception processes. However, other studies have shown that speech perception abilities might be compromised in PWS when acoustic stimuli are presented with limited cues [23–25]. For instance, AWS showed a lower speech recognition performance when they were required to detect vowel-consonant (VC) syllables in three conditions including the quiet condition, and the backward masking conditions when the maskers were presented in a 0 millisecond (ms) delay and a 300 ms delay with the onset of the speech syllable [26]. The authors hypothesized that this effect might be related to indistinct phonemic boundaries, especially when the acoustic stimuli were presented without lexical context or top-down semantic cues [26]. However, it was also suggested that the observed effect could be related to the forward masking exerted from a high energy vowel on a lower energy consonant in VC syllables rather than indistinct categorical boundaries [26]. Furthermore, auditory perception is shown to be compromised in non-speech stimuli using the backward masking paradigm in which CWS have a higher hearing threshold sensitivity when listening to pure tones followed by a masking noise compared with the CWNS [23] and children who had recovered from stuttering [24]. One study investigated the speech perception of the AWS compared with typically fluent speakers (TFS) using the categorical perception paradigm. The participants needed to identify the speech syllables (e.g. /be/ versus /pe/) that were systematically modified in terms of their voice onset time (VOT) continuum [27]. The results of the study revealed that the speech perceptual acuity measured by discriminatory power was less stable and the phoneme boundaries of the VOT continuum (e.g. /be/-/pe/) were placed at longer intervals in the AWS compared with the TFS [27]. The authors concluded that the phoneme representation in AWS is less stable or insufficiently accessed.

There are also some neurophysiological studies that examined the auditory perception of PWS using the oddball paradigm [28,29], in which the subjects listened to one tone (1 kHz) that was played frequently and a second tone (2 kHz) that was played infrequently. These studies reported no group differences in early ERP measures (e.g., N1 or P1) in adults and children who stutter compared to TFS [28,29]; however, CWNS, but not CWS, exhibited a significantly higher P3 amplitude when listening to infrequent tones compared to the frequent tones [29]. Based on these results, the authors suggested that deficiency in auditory attention and working memory function played a role in developmental stuttering [29]. Similar findings are also reported in ERP studies that used similar paradigms to examine the speech perception of PWS in which no group differences were found in the early ERP components (i.e., P1 and N1), whereas, the later ERP components such as mismatch negativity (MMN) were able to differentiate the PWS from TFS [30,31].

As discussed, these previous behavioral studies that examined the speech perception of PWS using stimuli that retained all their contextual cues (e.g., nonword repetition, phoneme monitoring, and phoneme deletion) resulted in inconsistent findings. The nonword repetition task fell short in disentangling the deficits in auditory perception, phonemic representation in speech perception, phonological encoding, and/or articulatory speech motor skills. Furthermore, the phoneme monitoring, and phoneme elision tasks might require further metalinguistic knowledge. However, the studies that examined the speech perception of PWS using acoustic stimuli with limited cues suggested a possible connection between speech perception deficits and stuttering. Thus, a cleaner paradigm that taps into phonemic representation deficits in speech perception without other potentially confounding factors such as contextual cues, articulatory speech motor skills and metalinguistic knowledge is warranted.

The current research aimed to study the speech perception of CWS compared to CWNS using the categorical perception paradigm to examine the CWS group’s ability to classify acoustic variations of a particular sound into either the same or different phonemic categories [32]. This method is a well-established measure that examines phonemic representations in children with developmental language and reading deficits [33–37]. Categorical perception is typically examined using identification and discrimination tasks. There is usually an abrupt response shift across categorical boundaries in identification tasks, while in discrimination tasks, differences between two stimuli from two different categories are easier to detect than two stimuli from the same category, even though their acoustic differences are equivalent [38,39].

To the best of our knowledge, only one study has investigated the speech perception of AWS using the categorical perception paradigm, which was limited to the VOT distinction of speech sounds [27]. However, since the previous study examined adults who stutter, the potential phoneme perception deficit in children remains unclear. To the best of our knowledge, categorical perception has not been directly studied in the CWS. Furthermore, the present study aimed to extend the current literature by examining categorical perception across three types of speech sound distinctions, including consonants (VOTs), lexical tones, and vowels in Cantonese using identification and discrimination tasks. We examined three types of speech sound distinctions in order to obtain a more comprehensive evaluation of the potential phonemic perception deficits in CWS. As our study was conducted in Cantonese, a tonal language that systematically distinguishes lexical meaning using pitch patterns, we designed a speech continuum that varied in suprasegmental distinctions (lexical tones), in addition to two speech continua of segmental distinctions (VOT for consonants and formant frequency for vowels) [40]. Note that the categorical perception of lexical tone distinctions has been used to distinguish the Chinese-speaking individuals with other types of speech perception deficits from typically developed individuals, such as developmental dyslexia [37,41] and congenital amusia [42–44].

## Materials and methods

### Participants

The participants included 15 CWS (12 males; age in months: 61–139; M = 100, SD = 20.59) and 16 CWNS (10 males; age in months: 62–132; M = 95.25, SD = 18.77) who spoke Cantonese as their native language. The two groups were matched in age, language ability (indicated by the textual comprehension subtest of the Hong Kong Cantonese Oral Language Assessment Scale, HKCOLAS, which examines the listening comprehension ability) [45], and nonverbal intelligence (indicated by Raven’s Standard Progressive Matrices) [46], as shown in Table 1. There were no reports of other known neurological/psychological diagnosis such as ADHD or autism in either group. The study was approved by the Human Subjects Ethics Committee of The Hong Kong Polytechnic University, and written consents were obtained from the parents of the CWS and the CWNS before commencement of the study. The families received monetary remuneration for their children’s participation in this study.

**Table 1:** Demographic information for the CWS and CWNS groups.

	CWS (15)			CWNS (16)			F	P-value
	Mean	SD	Range	Mean	SD	Range		
**Chronological Age (months)**	100	20.59	61–139	95.25	18.77	62–132	0.22	0.6
**HKCOLAS**	22.2	7.44	10–36	21	6.34	11–30	0.22	0.6
**RAVEN**	105.5	11.6	91–131	114.9	14.59	97–135	2.30	0.1
**%SS**	4.01	1.5	2–7	0	0	0	18.70	0

CWS = children who stutter; CWNS = children who do not stutter; SD = standard deviation; HKCOLAS = Hong Kong Cantonese Oral Language Assessment Scale (textual comprehension subtest); Raven = Raven’s Standard Progressive Matrices; %SS = percentage of syllables stuttered.

The initial diagnosis for the CWS was performed by a qualified speech therapist at a fluency clinic in the Speech Therapy Unit of The Hong Kong Polytechnic University. Two connected speech samples of at least 600 syllables, including conversational speech and storytelling speech, were collected. Stuttering severity was estimated by calculating the percentage of syllables stuttered (%SS) across the two speech samples based on SSI_3 [47]. To determine the interrater agreement of the %SS measures, about one-third of the speech samples were independently evaluated by two trained students with a Master of Science in Speech Therapy, and the high interrater agreements were achieved between the examiners (M = 0.87; range: 0.67–0.99).

### Stimuli

**Fig 1:**
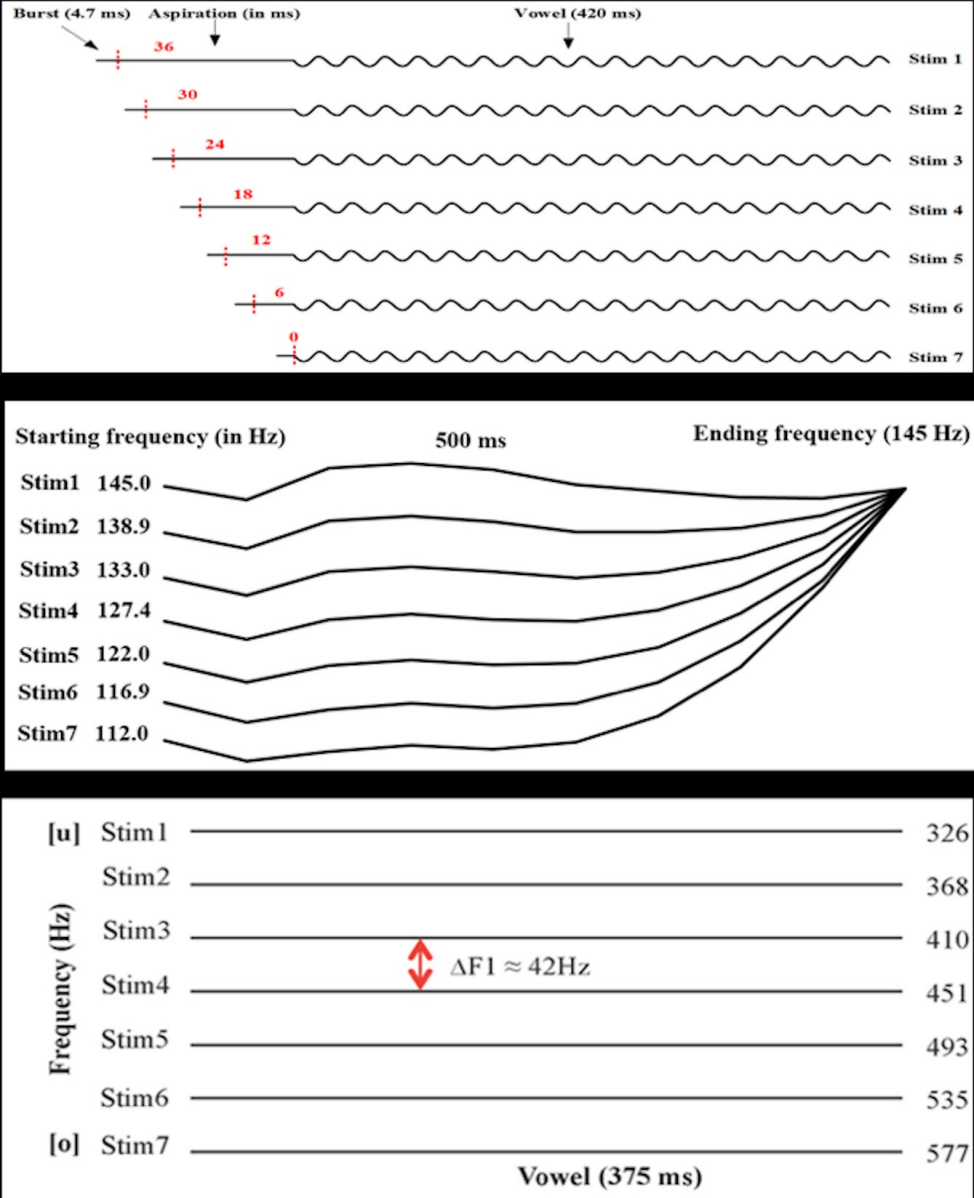
Schematic diagram of stimulus continua divided into seven stimuli (adopted from Zhang, Shao, & Huang, 2017). Top Fig: VOT continuum. Middle Fig: Lexical tone continuum. Bottom Fig: Vowel continuum.

Three types of stimulus continua—consonants (VOTs), lexical tones, and vowels—were constructed for this study following the methodology of Zhang, Shao, and Huang (2017) [44]. All three types of speech sound distinctions were included in order to fully assess phoneme representation in the CWS group compared with the CWNS group. Three pairs of minimally contrasted Cantonese words were chosen as the VOT, lexical tone, and vowel pairs, respectively: /pa55/ (疤 ‘scar’) and /p^h^a55/ (趴 ‘lying on one’s stomach’) for the VOT continuum; /ji55/ (醫 ‘doctor’) and /ji25/ (椅 ‘chair’) for the lexical tone continuum; and /fu55/ (膚 ‘skin’) and /fo55/ (科 ‘section’) for the vowel continuum. The lexical tones were annotated using Chao’s tone letters [44], which range from 1 to 5, with 5 indicating the highest pitch and 1 the lowest. Each tone was annotated with two digits, indicating in an abstract sense the pitch at the beginning and end of a syllable, respectively; for instance, /55/ indicates a high-level tone, while /25/ indicates a high rising tone. A male native Cantonese speaker read the selected words, which were recorded. The duration of all the selected words was normalized to 500 ms, and their mean intensity was normalized to 60 decibels (dB) using Praat [48] for further manipulation (Fig 1).

Referring to Fig 1, for the VOT continuum, following the initial normalization, the word /p^h^a55/ (趴 ‘lying on one’s stomach’) was used as the basis for manipulation and segmented into three sequential sections: burst release (~4.7 ms), aspiration (~36 ms), and vowel /a55/ (~420 ms). Then, the aspiration section was divided into seven steps that were proportionally shortened in duration by 6 ms (ΔVOT = 6 ms). Finally, each of the seven lengths in the aspiration section was concatenated with the preceding burst release and the following vowel to generate a continuum of seven equally spaced stimuli that varied in VOT between /pa55/ (疤 ‘scar’) and /p^h^a55/ (趴 ‘lying on one’s stomach’).

For the creation of the lexical tone continuum, fundamental frequency (F0) was measured at 11 time points at 10% intervals across the entire time course of the vowels in /ji55/ (醫 ‘doctor’) and /ji25/ (椅 ‘chair’), respectively. The F0 continuum, which reflected the F0 distance between /ji55/ and /ji25/ at each time point, was equally divided into seven steps in terms of semitones (ΔF0 ≈ 0.74 semitone) at the onset of the stimuli, which decreased toward the end of the stimuli. The syllable /ji55/ was used as the basis for pitch manipulation by replacing its original F0 contour with seven equally spaced F0 contours using Praat [45]. In this way, seven equally spaced pitch contours that varied on a continuum between /ji55/ and /ji25/ were generated.

For the creation of the vowel continuum, the two selected words /fu55/ (膚 ‘skin’) and /fo55/ (科 ‘section’) were segmented into two parts, the initial consonant (/f/) and vowel (/u/ or /o/). The frequencies of the first to fourth formants (F1-F4) were measured at 11 time points at 10% intervals across the entire duration of the vowels. Then, the smallest F1 value in the measurements of /u/ and the largest F1 value in the measurements of /o/ were selected as the two endpoints of the F1 continuum, which was then equally divided into seven steps in hertz (Hz). Furthermore, the mean F2-F4 frequencies of /u/ and /o/ were calculated and used to synthesize the stimuli. Then, using the vowel /u55/ as the basis for manipulation, seven stimuli were synthesized by setting the frequencies of F1-F4 to the designated values in seven steps using Praat [45]. Finally, the seven vowel stimuli were concatenated with the preceding consonant /f/, generating a continuum of seven equally spaced stimuli that varied in F1 between /fu55/ and /fo55/, with the frequencies of F2-F4 kept constant across the seven stimuli.

### Procedure

Each stimulus continuum was presented in an identification task and a discrimination task using the E-prime 2 software. For the identification task, each stimulus continuum (i.e., VOT, lexical tone, and vowel) was presented in a separate block in which the seven steps of the continuum (stimuli 1–7) were repeated in random order eight times, resulting in a total of 56 randomly ordered trials. In each trial, an auditory stimulus was presented to the participants binaurally through headphones, and they were instructed to identify the stimulus by pressing the corresponding button on the keyboard labeled with the Chinese characters /pa55/ (爸 ‘father’) and /p^h^a55/ (趴 ‘lie down’) for the VOT condition, /ji55/ (衣 ‘clothing’) and /ji25/ (椅 ‘chair’) for the lexical tone condition, and, finally, /fu55/ (膚 ‘skin’) and /fo55/ (科 ‘subject’) for the vowel condition within five seconds.

For the discrimination task, again, each stimulus continuum was presented in a separate block. In each stimulus continuum, a total of 12 pairs were created for discrimination, with seven identical pairs (i.e., stimuli pairs 1–1, 2–2, 3–3, 4–4, 5–5, 6–6, and 7–7) and five different pairs that were separated by two steps (i.e., stimuli pairs 1–3, 2–4, 3–5, 4–6, and 5–7). The interstimulus interval (ISI) of the two stimuli in each pair was 500 ms. While the seven identical pairs were repeated five times (35 trials), the five different pairs were repeated seven times (35 trials), generating a total of 70 randomly ordered pairs for each continuum, with an equal number of same and different pairs. The auditory stimuli were presented to the participants binaurally through headphones, and they were instructed to discriminate whether the pair of stimuli sounded identical or different by pressing different buttons on the keyboard (the left arrow for “same” responses and the right arrow for “different” responses). The accuracy data and reaction times, which were measured from the offset of the stimuli, were measured across both tasks.

The identification task (including all three stimulus continua) preceded the discrimination task (including all three stimulus continua). Before each task, a practice block, which contained the same types of stimuli as in the first experimental block, was given to the participants to familiarize them with the procedure. In the practice identification task, the seven stimuli in the continuum were presented only once in random order. In the practice discrimination task, 15 practice trials comprised of four different pairs of stimuli separated by three steps (i.e., stimuli pairs 1–4, 2–5, 3–6, and 4–7) in forward and reverse orders (eight trials) and seven identical pairs (seven trials) were randomly presented.

### Data analysis

#### Identification task

For the identification task, an identification curve was made for each participant, and probit analysis was applied to obtain the value of the boundary position and width for each stimulus continuum [49]. The boundary position represented the position of the perception shift across two categories and the boundary width represented the steepness of the response shift across categorical boundaries. The sharper the slope, the more categorical the perception. The boundary position was estimated as the 50% crossover point in a continuum, while the boundary width was obtained as the distance between the location of 25% and 75% of the responses along the stimulus continuum in the probit analysis [50]. For instance, if 25% of stimulus 1 and 75% of stimulus 6 were identified as /p^h^a55/ (趴 ‘lying on one’s stomach’) in the VOT continuum, the boundary width would be 5 (6–1 = 5).

Following the probit analysis, some data from a few participants had to be discarded from further analysis for each continuum since no reliable boundary position could be calculated from their identification curves (e.g., the boundary position was a negative value or larger than the maximal stimulus, step seven). Overall, the boundary position and width were calculated for 13 CWS and 14 CWNS in the VOT and vowel conditions and for 14 CWS in the lexical tone condition.

### Discrimination task

For the discrimination task, sensitivity index d' was used for data analysis [51]. The d' was formulated by subtracting the z-score of the hit rate (different responses to “different” pairs) from that of the false alarm rate (different responses to “identical” pairs) for pairs in each stimulus continuum per participant. For instance, for the pair 4–6, the hit rate was the average of correct responses to “different” pairs of 4–6 and 6–4, whereas the false alarm rate was the average of incorrect responses to “identical” pairs of 4–4 and 6–6. Furthermore, for each participant, the pairs were divided into between-category and within-category groups according to their boundary position obtained from the identification task. For example, if the boundary position was 4.5, then pairs 3–5 and 4–6 were placed in the between-category group, while the remaining pairs 1–3, 2–4, and 5–7 were placed in the within-category group.

### RT Data

The RT data were collected using the E-prime 2 software to investigate the speed of perceptual processing in two tasks. For the analysis of the RT data, initially, RT responses below 200 ms were removed from further analysis in both tasks, which resulted in 15% of data reduction. Furthermore, the RT data from the identification task were classified into two groups—between-category and within-category—based on the boundary position obtained from the probit analysis for each participant across the seven steps of stimuli for each stimulus continuum. For instance, if the boundary position for a particular participant was 3.5, then stimuli 3 and 4 were placed in the between-category group and the rest of the stimuli (i.e., 1, 2, 5, 6, and 7) were placed in the within-category group.

A linear mixed effect (LME) model was used for analysis. Participants and stimuli pairs as random effects were used to analyze the data for the boundary position, boundary width, and RTs in the identification task and for the d' analysis and RTs in the discrimination task. LME modeling has become standard in psycholinguistic research [52,53], as the modeling of item and participant factors as random effects increases generalizability by considering the variability across the items and participants, allowing population level inferences to extend beyond a limited number of participants and items in experiments [53].

## Results

### Identification task

**Fig 2:**
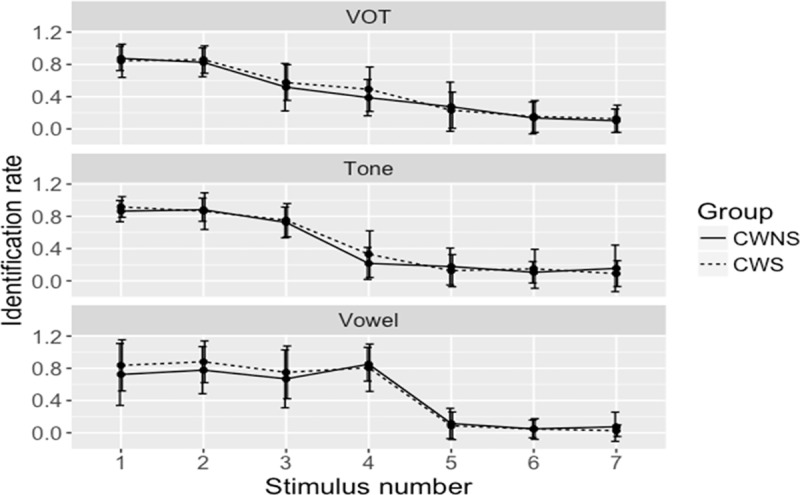
Response curves for the CWS and CWNS groups across the three stimulus continua in the identification task (Error bars: +/- 1 SE). Top Fig: Rate of /pa55/ (爸 ‘father’) responses in the VOT continuum. Middle Fig: Rate of /ji55/ (衣 ‘clothing’) responses in the lexical tone continuum. Bottom Fig: Rate of /fu55/ (膚 ‘skin’) responses in the vowel continuum.

**Fig 3:**
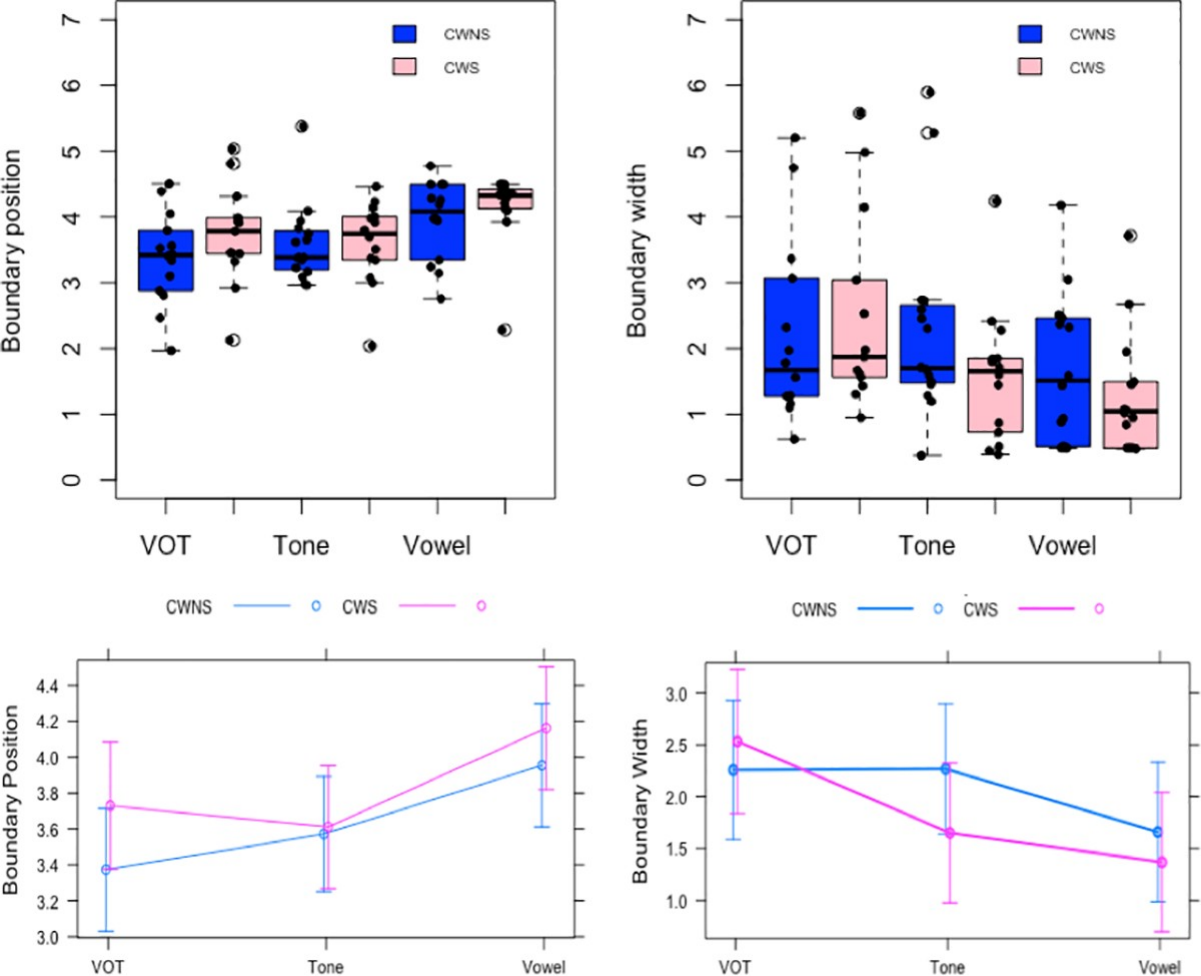
Boundary position and width for the CWS group compared with the CWNS group across the three stimulus continua in the identification task. Top Figs: Individual scores. Bottom Figs: Interaction plots.

The boundary position and width were analyzed using the LME model, with the groups (CWS and CWNS) and the stimulus continua (VOT, vowel, and lexical tone) as fixed effect and the participants as random effect variables. The results revealed no significant differences between the CWS and CWNS groups in terms of boundary position (Estimate = 0.2, Std. Error = 0.24, *t* = 0.85, *p =* 0.4). In terms of the stimulus continua, the pairwise comparison with Tukey adjustment showed that the boundary position for vowels was significantly higher than for VOTs (Estimate = 0.51, Std. Error = 0.17, *t* = 2.9, *p* = 0.013) and lexical tones (Estimate = 0.46, Std. Error = 0.17, *t* = -2.76, *p* = 0.021), but no significant differences were found between VOTs and lexical tones (Estimate = 0.40, Std. Error = 0.17, *t* = 0.23, *p* = 0.96). Moreover, there was no significant interaction between the groups and the stimulus continua. Regarding boundary width, the LME analysis revealed no significant differences between the CWS and CWNS groups (Estimate = -0.29, Std. Error = 0.47, *t* = -0.61, *p* = 0.54). In terms of the stimulus continua, the pairwise comparison with Tukey adjustment showed only one significant difference in which the boundary width for vowels was significantly lower than for VOTs only (Estimate = -0.88, Std. Error = 0.26, *t* = -3.28, *p* = 0.005). Furthermore, the interactions between the groups and the stimulus continua was not significant. The identification curves of the VOTs, lexical tones, and vowels across the seven stimuli steps for the CWS group compared with the CWNS group are shown in Fig 2, and the boundary position and width across the three stimulus continua for the CWS group compared with the CWNS group are shown in Table 2 and Fig 3.

**Table 2:** Descriptive data for the boundary position and width in the identification task.

		Boundary Position		Boundary Width			
Group	Stimuli Pairs	Mean	SD	Range	Mean	SD	Range
**CWNS**	Tone	3.57	0.59	2.96–5.38	2.27	1.45	0.38–5.89
**CWS**	Tone	3.61	0.63	2.04–4.46	1.58	1.02	0.39–4.24
**CWNS**	VOT	3.37	0.70	1.97–4.51	2.20	1.40	0.62–5.20
**CWS**	VOT	3.73	0.76	2.13–5.03	2.51	1.49	0.95–5.58
**CWNS**	Vowel	3.96	0.61	2.76–4.78	1.69	1.15	0.49–4.18
**CWS**	Vowel	4.16	0.57	2.28–4.50	1.30	0.93	0.48–3.71

**Fig 4:**
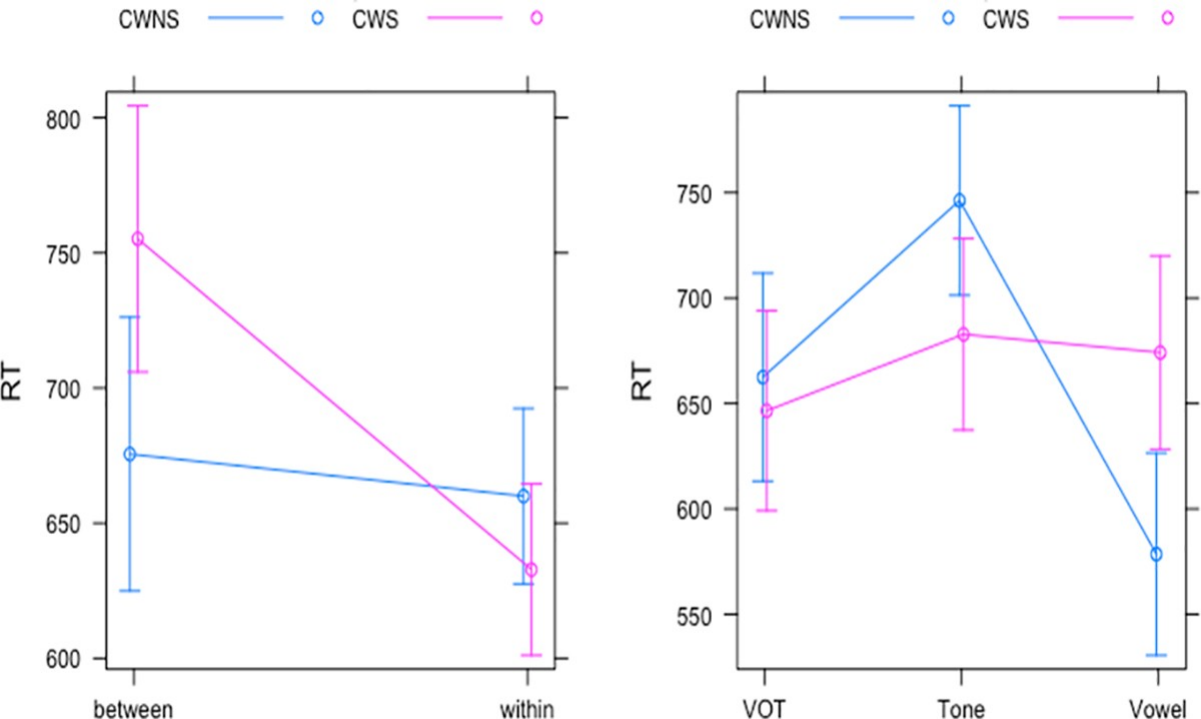
Interaction effect plots for the RTs of the CWS and CWNS groups across the categorical boundaries (right) and three stimulus continua (left) in the identification task.

For the analysis of the RTs, an LME analysis was conducted, with the groups (CWS and CWNS), categories (between-category and within-category), and stimulus continua (VOT, vowel, and lexical tone) as fixed effect variables and the participants and stimuli pairs as random effect variables (Table 3). The results showed a significant main effect of the stimulus continua, but no significant effects of the categories and groups (Table 4). In terms of interactions, the groups and stimulus continua interaction showed that the CWS group was significantly slower than the CWNS group in the vowel condition only (mean RT of the CWS group: 655 ms, SD: 601 and the CWNS group: 601 ms, SD: 492). The groups and categories interaction indicated that the CWS group was significantly slower in the identification of between-category stimuli (mean RT: 747 ms, SD: 648) compared with within-category stimuli (mean RT: 628 ms, SD: 555), while the CWNS group did not show any significant difference between the two categories (mean RT of between-category: 688 ms, SD: 604 and within-category: 668 ms, SD: 656) (Fig 4).

**Table 3:** RTs for the identification and discrimination tasks.

			Identification Task			Discrimination Task		
Group	Stimuli Pairs	Category	Mean	SD	Range	Mean	SD	Range
CWNS	Tone	Between	781	695	209–4330	734	476	206–2707
CWNS	Tone	Within	740	775	200–4937	744	514	203–2797
CWNS	VOT	Between	630	584	204–4750	723	509	202–2786
CWNS	VOT	Within	686	643	203–4612	769	572	201–2869
CWNS	Vowel	Between	645	477	204–3150	729	425	207–2262
CWNS	Vowel	Within	585	497	202–4167	761	515	202–2997
CWS	Tone	Between	775	572	202–4063	904	544	204–2662
CWS	Tone	Within	634	528	201–4957	892	536	213–2724
CWS	VOT	Between	803	713	211–4374	773	522	229–2784
CWS	VOT	Within	602	556	203–4846	849	625	204–2977
CWS	Vowel	Between	670	657	204–4057	911	581	201–2850
CWS	Vowel	Within	652	579	200–3834	894	605	201–2950

**Table 4:** LME analysis of the RTs of the CWS and CWNS groups in the identification task.

**Fixed Effects:**	**Estimate**	**Std. Error**	**df**	**t value**	**p-value**	
**(Intercept)**	673.440	58.140	56.740	11.583	< 2e-16	***
**GroupCWS**	59.680	80.420	52.280	0.742	0.461	
**ConditionTone**	83.810	33.000	3923.600	2.539	0.011	*
**ConditionVowel**	-83.900	34.620	3931.560	-2.424	0.015	*
**CategoryWithin**	-15.560	31.860	203.660	-0.488	0.626	
**CWS:ConditionTone**	-47.570	46.650	3930.870	-1.020	0.308	
**CWS:ConditionVowel**	111.510	47.900	3930.860	2.328	0.020	*
**CWS:CategoryWithin**	-106.820	40.980	3847.790	-2.606	0.009	**
**Random Effects:**		**Variance**	**Std. Dev.**			
**Participants**	(Intercept)	34508.20	185.76			
**Stimuli Pairs**	(Intercept)	771.10	27.77			
**Residual**		339242.40	582.45			

Significance

***0.001

**0.01

*0.05

Sta. Error = standard error; df = degree of freedom; Std. Dev. = standard deviation.

### Discrimination task

**Fig 5:**
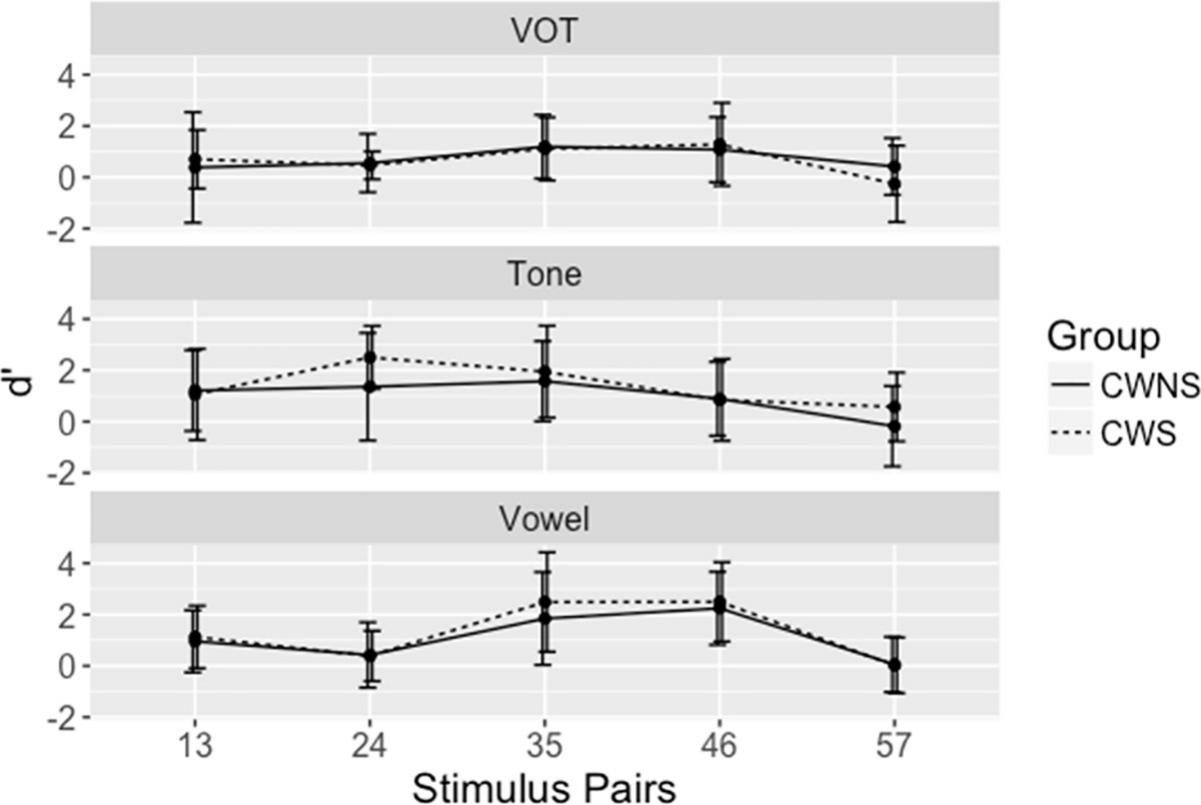
The d' scores of each stimulus continuum for the CWS and CWNS groups in the discrimination task (Error bars: +/- 1 SE).

**Fig 6:**
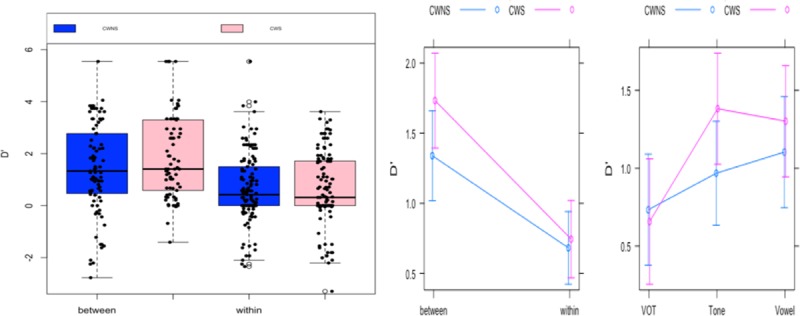
The d' scores (left) and interaction plots (right) across the categorical boundaries and stimulus continua for the CWS and CWNS groups in the discrimination task.

LME analysis was conducted to compare the d' scores across the groups (CWS and CWNS), stimulus continua (VOT, vowel, and lexical tone), and categories (between-category and within-category). The results revealed a significant effect of categories only (Estimate = -0.65, Std. Error 0.23, *t* = -2.9, *p* < 0.01), as the d' scores of the between-category pairs were significantly higher than those of the within-category pairs, providing evidence for categorical perception (Table 5). No other effects were significant. The d' scores across different stimuli pairs and categories are depicted in Figs 5 and 6, respectively.

**Table 5:** LME analysis of the D-prime values of the CWS and CWNS groups in the discrimination task.

Fixed Effects:	Estimate	Std. Error	df	t value	p-value	
**(Intercept)**	1.123	0.290	18.264	3.869	0.001	**
**GroupCWS**	0.128	0.327	231.585	0.391	0.696	
**CategoryWithin**	-0.656	0.229	321.021	-2.866	0.004	**
**ConditionTone**	0.240	0.241	386.855	0.995	0.320	
**ConditionVowel**	0.383	0.250	399.859	1.532	0.126	
**CWS:CategoryWithin**	-0.331	0.298	371.152	-1.109	0.268	
**CWS:ConditionTone**	0.486	0.359	392.815	1.352	0.177	
**CWS:ConditionVowel**	0.263	0.365	398.337	0.722	0.471	
**Random Effects:**		**Variance**	**Std. Dev.**			
**Participants**	(Intercept)	0.022	0.150			
**Stimuli Pairs**	(Intercept)	0.159	0.399			
**Residual**		2.169	1.473			

Significance

**0.01.

**Fig 7:**
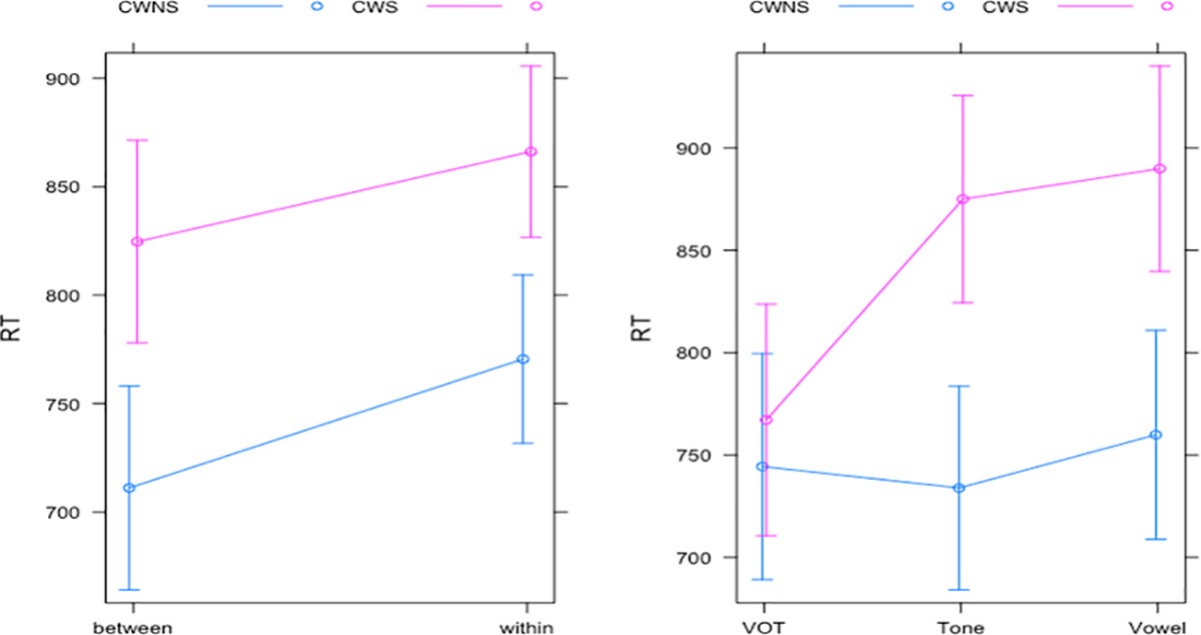
Interaction plots for the RTs of the CWS and CWNS groups across the categorical boundaries (left) and stimulus continua (right) in the discrimination task.

For the analysis of the RTs, LME analysis was conducted, with the groups (CWS and CWNS), stimulus continua (VOT, vowel, and lexical tone), and categories (between-category and within-category) as fixed effect variables and the participants and stimuli pairs as random effect variables in the discrimination task (Table 3). The results showed no main effects of the groups and stimulus continua, but a trend for a significant effect of categories (i.e., a trend for slower RTs for the within-category pairs versus the between-category pairs). However, a significant interaction between the groups and stimulus continua was found in which the RTs in the CWS group were significantly slower in the vowel (mean RT of the CWS group: 901 ms, and the CWNS group: 747 ms) and lexical tone (mean RT of the CWS group: 897 ms, and the CWNS group: 740 ms) conditions, but not in the VOT condition (mean RT of the CWS group: 818 ms, and the CWNS group: 750 ms), as shown in Tables 3 and 6 and Fig 7. Furthermore, there was no significant interaction between the groups and the categories, indicating that the RTs for the between-category pairs versus the within-category pairs were comparable across the CWS and CWNS groups (Table 6 and Fig 7).

**Table 6:** LME analysis of the RTs of the CWS and CWNS groups in the discrimination task.

Fixed Effects:	Estimate	Std. Error	df	t value	p-value	
**(Intercept)**	709.450	67.710	44.780	10.477	0.000	***
**GroupCWS**	33.220	93.010	42.620	0.357	0.723	
**CategoryWithin**	59.340	31.690	738.720	1.873	0.062	.
**ConditionTone**	-10.470	35.300	2464.570	-0.297	0.767	
**ConditionVowel**	15.510	36.490	2477.500	0.425	0.671	
**CWS:CategoryWithin**	-17.830	40.920	2447.010	-0.436	0.663	
**CWS:ConditionTone**	118.400	50.790	2467.670	2.331	0.020	*
**CWS:ConditionVowel**	107.290	51.570	2474.780	2.080	0.038	*
**Random Effects:**		**Variance**	**Std. Dev.**			
**Participants**	(Intercept)	48938	221.22			
**Stimuli Pairs**	(Intercept)	2147	46.34			
**Residual**		247174	497.17			

Significance

**0.01

*0.05,. = 0.1.

Moreover, in order to see whether the slower RTs in the CWS group could be explained by the speed-accuracy trade-off, another LME analysis was conducted, with RTs as a dependent variable and response accuracy and groups as the fixed effect variables. The results indicated a significant main effect of accuracy on RTs (Estimate = 144.07, Std. Error = 28.78, *t* = 5.01, *p* < 0.001), meaning that the higher the accuracy, the slower the RTs. However, there was no significant interaction between the groups and accuracy (Estimate = -44.65, Std. Error = 40.88, *t* = -1.09, *p* = 0.275), indicating that the slower RTs for the CWS group compared with the CWNS group could not be explained by the speed-accuracy trade-off.

Finally, as the groups were not completely matched based on the gender difference, two LME models, one with the gender variable and one without the gender variable, were conducted and the comparison between the two models showed that the gender factor cannot explain the RT difference between the two groups neither in the identification task (χ2(1) = 1.37, *p* = 0.24), nor in the discrimination task (χ2(1) = 0.24, *p* = 0.63).

## Discussion

The purpose of this study was to identify any speech perception differences between Cantonese-speaking CWS and CWNS by examining the categorical perception of three types of speech sound distinctions (i.e., VOTs, lexical tones, and vowels). The results showed that there were no significant differences between the CWS and CWNS groups in terms of the boundary position and boundary width across the three stimulus continua in the identification task. Regarding the discrimination task, although the overall patterns indicated that the subjects’ perceptions were categorical (i.e., higher d' scores for the between-category pairs compared with the within-category pairs), again, no significant difference was found between the CWS and CWNS groups with regard to the benefit of between-category discrimination relative to within-category discrimination (Fig 6). These results suggest similar capacities in categorical perception between the CWS and CWNS groups and refute a robust deficit in the organization of phonemic representation measured in speech perception among the CWS. In contrast, a former study [27] that used a similar paradigm found that the phonemic boundaries were located at a longer VOT continuum for AWS compared with TFS. Although our study found a similar pattern (i.e., a higher phonemic boundary position and width in the VOT continuum for the stuttering group), the difference was not statistically significant. One important difference between the two studies is the target age group, as the former study examined AWS and the current study focused on CWS. As argued by others, there are some differences between adults and children in terms of their attention capacities toward auditory stimuli and the time course of auditory perception development [26] that might have played a role in the different findings in the two studies.

However, the findings in the current study did reveal significant differences in RTs between the CWS and CWNS groups. In the identification task, the CWS group was generally slower than the CWNS group, which was statistically significant for the vowel condition (Fig 4). Similar results were found in the discrimination task, where the RTs were significantly slower in the CWS group than in the CWNS group across the lexical tone and vowel conditions (Fig 7). This suggests that although the CWS group showed a comparable performance in the categorical perception of the speech stimuli, implying more or less intact phonemic representation in speech perception, the processing speed of speech perception might have been compromised in the CWS group. It is notable that the former categorical perception study [27] did not report any RT data; therefore, our study is the first to report processing speed differences in categorical perception in CWS compared with CWNS.

Another important finding of this study is that the CWS group was slower in the identification of stimuli that were placed across the boundary position (between-category) compared with the stimuli placed further away from the boundary (within-category) (Fig 4). Stimuli located around the categorical boundary were usually more ambiguous than stimuli located further away from the boundary, which suggests that this ambiguity might have imposed a higher perceptual processing demand on the CWS group when categorizing the acoustic stimuli into the corresponding phonemic categories. This result resonates with studies that have reported a poorer speech recognition ability for PWS in challenging listening situations, such as speech perception under the backward masking condition (i.e., when a masking noise is presented subsequently after the speech sound) compared with the quiet condition [23–26]. It has been suggested that backward masking either interferes with the temporal processing of the speech sound or erases its relevant memory [26]. It is possible that the slower RTs in the CWS group’s identification of between-category versus within-category stimuli implies that the cognitive process involved in the transition and establishment of ambiguous acoustic stimuli in the phonemic category was more effortful for the CWS.

Unlike the identification task, the processing time for the discrimination of between-category and within-category stimuli in the CWS group was not significant (Fig 7). One explanation for this discrepancy is that the identification of speech stimuli required more in-depth perceptual processing and explicit access to phonemic representation during speech perception compared with the discrimination of speech stimuli, which can be accomplished with less in-depth acoustic analysis of the stimuli. This interpretation is partly in line with event-related potential (ERP) studies on auditory perception in CWS, which did not find any significant differences between the CWS and CWNS groups regarding the amplitudes and latencies of the early ERP components (i.e., P1 and N1) that were more related to the initial stages of sound feature analysis [29,31]. However, later ERP components such as MMN and P300, which were related to more in-depth perceptual processing such as stimulus probability, quality, duration, working memory and access of long-term phonemic representations, were able to differentiate CWS and AWS from TFS [28–31].

As discussed earlier, previous studies that investigated phonemic representations in speech perception used different paradigms, including those that presented acoustic stimuli with high and low contextual cues. The studies that used stimuli with high contextual cues (e.g., in nonword repetition and phoneme monitoring tasks) reported some mixed findings [16]. The current study used a different paradigm for the investigation of phonemic representations evaluated in speech perception (i.e., categorical perception), which showed that, in general, the organization of phonemic representation was not compromised in the CWS group; however, the CWS group was slower in perceptual speech processing, especially when listening to ambiguous stimuli located across categorical boundaries. This might indicate that CWS need more time to access phoneme representation in speech perception in order to achieve an identical level of categorical perception as that of CWNS.

As discussed, the CWS group showed a similar pattern of categorical perception compared with the CWNS group in terms of boundary position and width in the identification task and d' values in the discrimination task. However, the lack of robust differences between the two groups can be explained by several factors. First, it was notable that our stuttering participants had a relatively mild stuttering severity (Table 1). Examining children with a higher stuttering severity might result in an increased effect size. Second, in contrast to the earlier study [27], which focused on AWS, our study focused on children’s categorical perception. It has been argued that children’s data are usually more varied than adult data, as children are usually less attentive to the acoustic stimuli and possess different developmental trajectories regarding the formation of auditory perceptual abilities [26,54–58]. Therefore, future studies should compare the auditory perception of children and adults with a higher stuttering severity using a similar speech perception paradigm.

Furthermore, this study found that the CWS group had slower RTs compared with the CWNS group. It has been argued that slower RTs in PWS can account for other differences, such as motor coordination, or cognitive functions, such as attention or executive functions [27,59]. Although a comparable manual button-press RT in response to auditory stimuli (as opposed to a laryngeal RT) has been reported between AWS and adult who do not stutter (AWNS) [60], future studies might benefit from the inclusion of a choice button-press RT task to rule out any possible effect of hand motor coordination on RTs in children. Furthermore, although we tested the cognitive ability of the participants using Raven’s Standard Progressive Matrices [46], which requires certain levels of attention and executive control, future studies should use other specific tasks such as a go/no-go task to match the attention and executive skills of CWS and CWNS.

## Conclusion

Overall, the findings from this study may not fully support the presence of a robust deficit in the organization of phonemic representation evaluated in speech perception (i.e., categorical perception) in CWS; however, it supports the presence of slower perceptual speech processing and difficulty in accessing phonemic representation in a timely manner, especially when the acoustic stimuli were more ambiguous. Given the fact that the behavioral measures mainly accounted for the endpoints of the cognitive processes, one future direction could involve the use of ERPs, which offer a high temporal resolution in examining the online cognitive processes of speech perception in CWS.

## Supporting information

S1 File The individual data points for boundary position and width in the CWS and CWNS groups. (XLSX)

S2 File The individual data points for d' scores in the CWS and CWNS groups. (XLSX)
